# Supplementary material for: Anion-exchange chromatography mass spectrometry provides extensive coverage of primary metabolic pathways revealing altered metabolism in IDH1 mutant cells
Source: Commun Biol. 2020 May 20;3:247. doi: 10.1038/s42003-020-0957-6 (PMC7239943; doi:10.1038/s42003-020-0957-6)
Supplement: Supplementary file 1 — Supplementary Information [file 42003_2020_957_MOESM1_ESM.pdf]

Supplementary Figures

Anion-exchange chromatography mass spectrometry (IC-MS) provides extensive coverage of primary metabolic pathways revealing altered metabolism in IDH1 mutant cells.

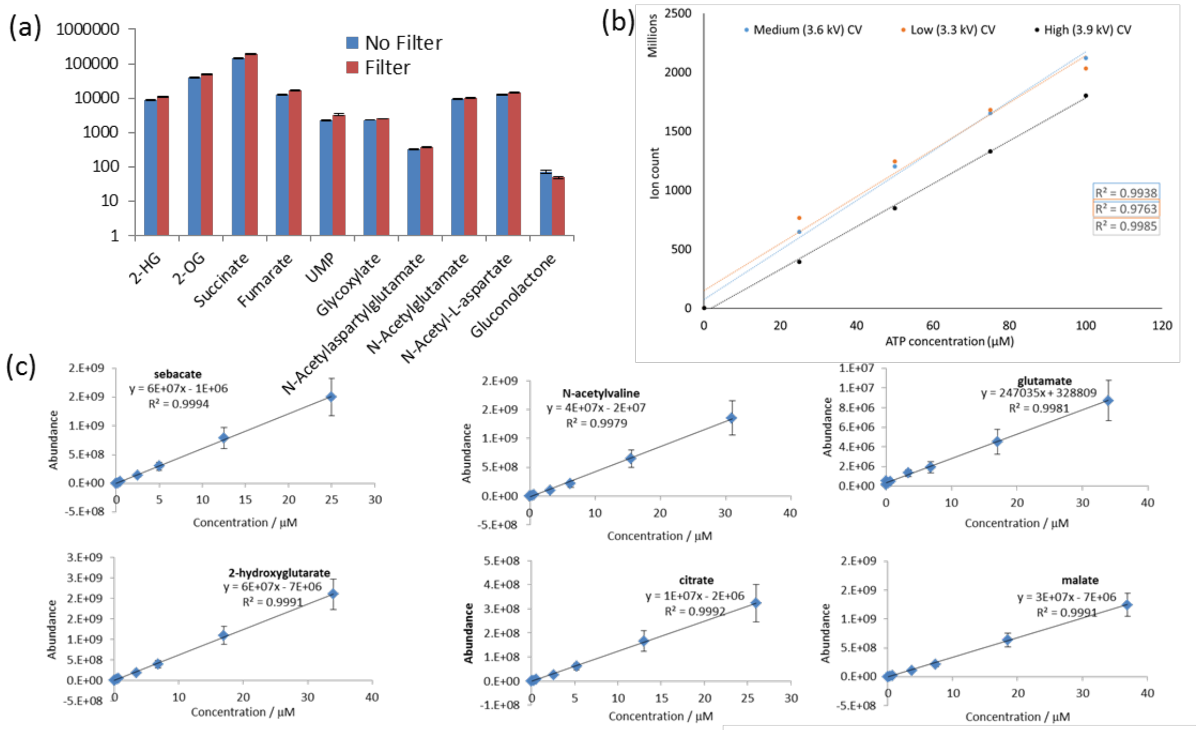

(d)

Metabolite	Compound type	LLOD (nM)	Linearity (R2)	Rt. Range (secs over 3 months)
N-Acetylphenylalanine	Acetylated amino acid	2.4	0.999	5.9
Guanosine monophosphate	Nucleotide	1400	0.900	8.4
Glyceraldehyde-3-phosphate	Mono-alkylphosphate	30	0.900	5.3
Phosphoenolpyruvate	Phosphate ester	600	0.999	7.3
Glutamate	Amino acid	34	0.998	5.8
Isovalerylglycine	Fatty acid derivative	3.1	0.999	8.6
Succinate	Dicarboxylic acid	0.42	0.990	7.6
Malate	Dicarboxylic acid	0.37	0.999	12.6
Citrate	Tricarboxylic acid	2.6	0.999	7.4
Fumarate	Dicarboxylic acid	4.4	0.990	7.2
2-hydroxyglutarate	Hydroxy-dicarboxylic acid	0.2	0.999	5.1
Sebacate	Dicarboxylic acid	2.5	0.999	7.7
Methylglutarate	Hydroxy acid	0.34	0.990	6.4
N-acetylvaline	Acetylated amino acid	0.31	0.998	7.2

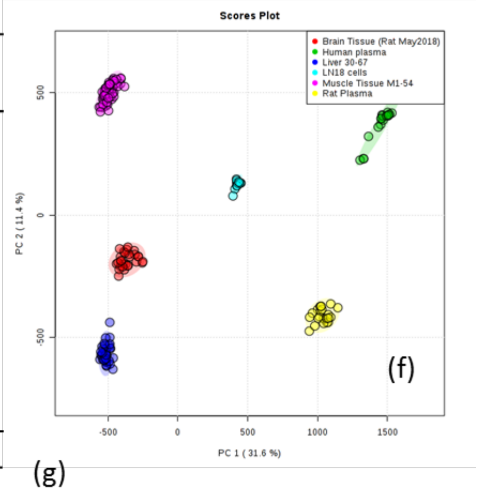

(e)

LC-MS type	Total compounds features	Compounds features with %CV <15	% of total compounds with %CV<15	Compound features with %CV <30	% of total compounds with %CV<30
IC-MS	5144	1386	27	2167	42
HILIC-MS (-ve)	3409	884	26	1591	47
Reversed-phase (-ve)	5204	2827	54	4187	80

(g)

Sample type	Compound features	%CV <30
Brain tissue (Rat)	5666	1086
Plasma (Human)	3567	7
Liver tissue (Rat)	6188	797
LN18 cell line (Human)	5144	2167
Muscle tissue (Rat)	5979	1022
Plasma (Rat)	5063	731

Supplementary Figure. 1: Targeted and untargeted IC-MS/MS method validation. (a) Bar graph comparing the ion abundance for selected metabolites before and after filtering with a 10kD molecular weight cut-off filter (Amicon, Merck) showing negligible changes in the metabolite abundance. The Y-axis is a log-scaled ion abundance. Error bars represent standard deviation of 9 analytical replicates per metabolite. **(b)** ATP shows a linear response for ion count plotted against concentration at different cone voltages which induce different amounts of phosphate loss from ATP. These data, and additional experiments on other multiple phosphorylated metabolites (data not shown), demonstrate in-source fragmentation has negligible impact on relative quantification. **(c)** Linearity for selected metabolite standards using IC-MS/MS indicated by R^2 values (n=3, error bars show S.D). **(d)** Table summarising method validation measurements for selected metabolite standards. Retention time variability (in seconds) is SD of 10 replicates over 3 month period. **(e)** Table compares total compound features with those having a %CV value <15 and <30 for HILIC-MS, C18 reversed-phase-MS and IC-MS. The table provides numbers of compound features measured by three different LC-MS methods from the same LN18 cell extract sample (N=9). **(f)** Principal component analysis plot showing clustering of samples into their sample type representing human and animal cells, tissues and bio-fluid samples. **(g)** Table showing the number of compound features measured by IC-MS in the analysis of various types of biological sample extracts.

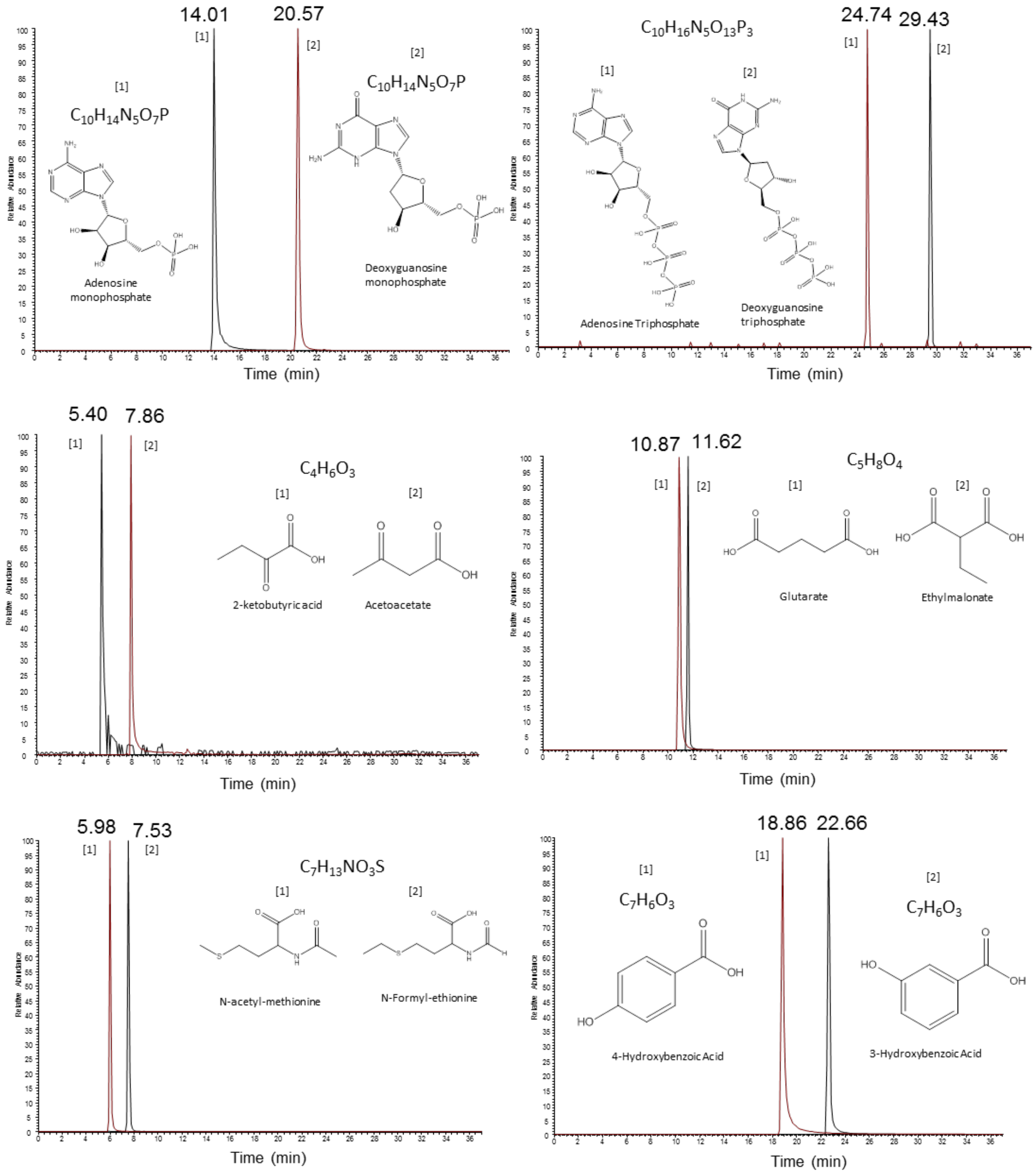

Supplementary Figure. 2: Extracted ion chromatograms (EICs) for selected metabolic structural isomers resolved using anion chromatography coupled to mass spectrometry.

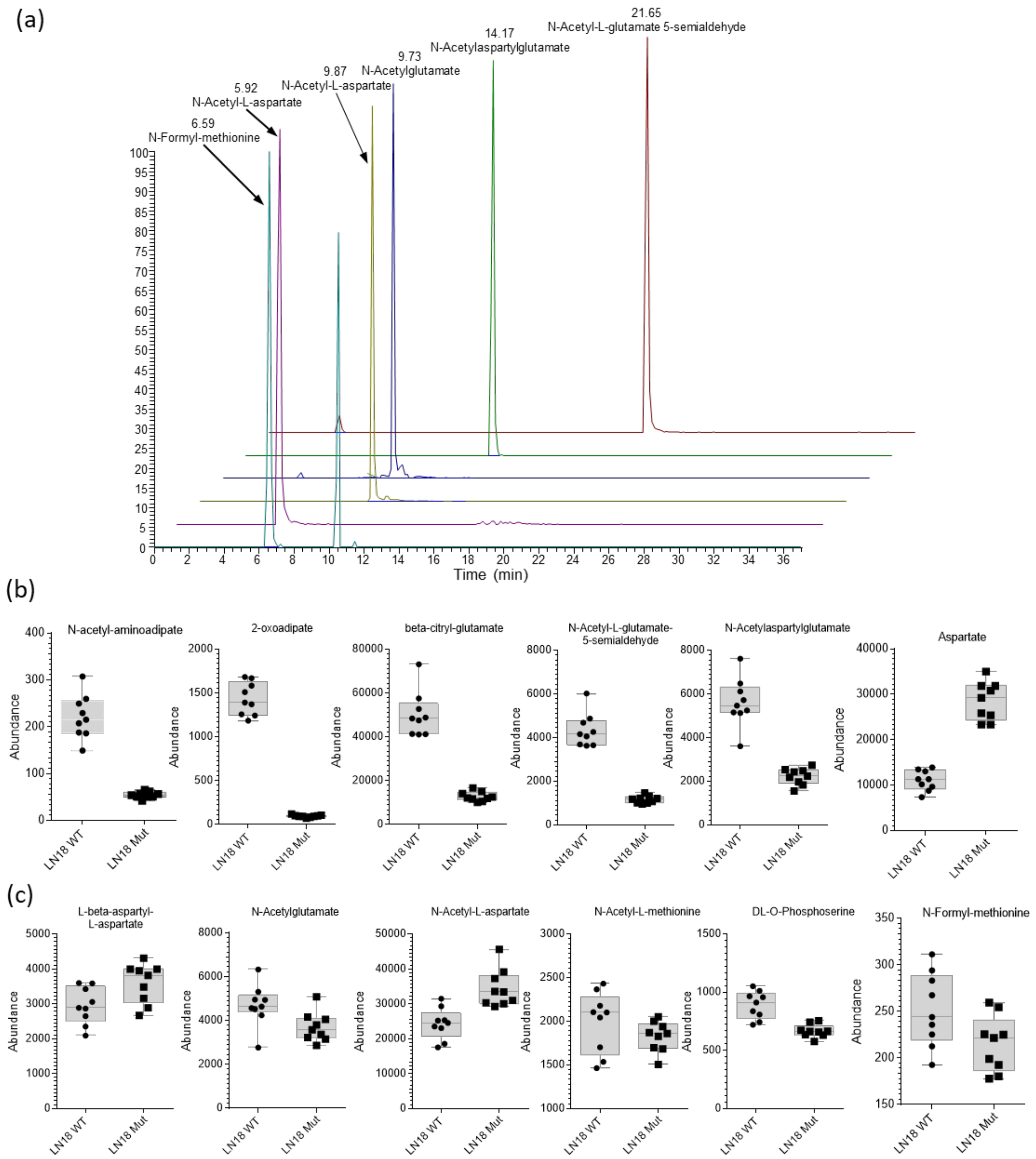

Supplementary Figure. 3: Normalised amino acid-derived compounds comparing IDH WT vs IDH MT cells. (a) EICs for selected amino acid-derived metabolites measured by IC-MS in LN18 IDH1 mutant and wild type cells. **(b)** Box plots showing significantly altered amino acid-derived metabolites in LN18 IDH1 mutant vs wild type cells. **(c)** Box plots representing abundances of additional amino acid-derived metabolites measured by IC-MS that were not altered between LN18 IDH1 mutant vs wild type cells. n=9 per experimental group. Box extends from the 25th to the 75th percentile with the median shown by the line in the middle. Whiskers are min to max with all data points shown.

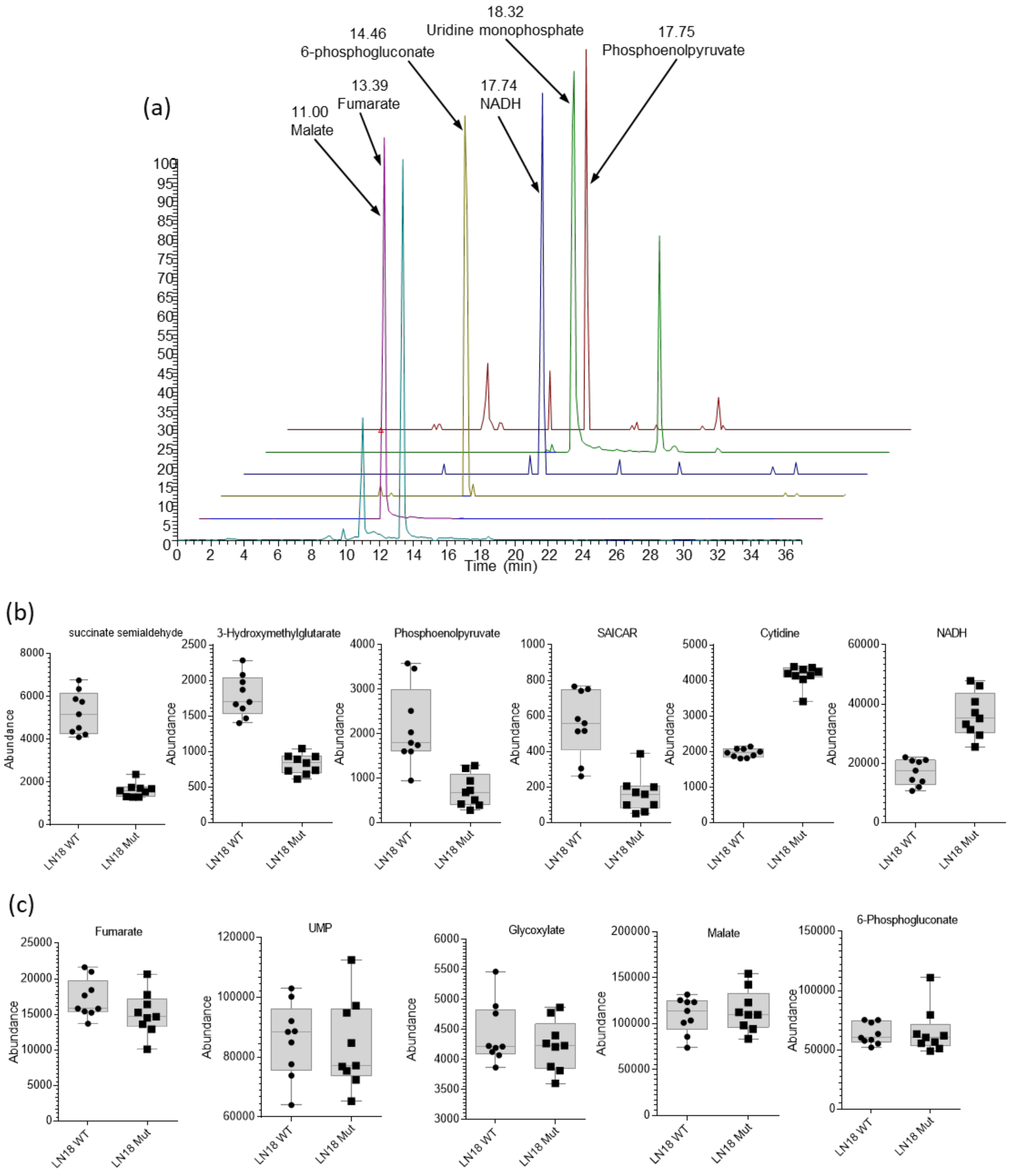

Supplementary Figure 4: Selected metabolites identified in IDH1 mutant cells. (a) EICs for selected metabolites measured by IC-MS in LN18 IDH1 mutant and wild type cells. **(b)** Box plots showing significantly altered non amino acid-derived metabolites in LN18 IDH1 mutant vs wild type cells. **(c)** Box plots representing abundances of additional metabolites measured by IC-MS that were not altered between LN18 IDH1 mutant vs wild type cells. n=9 per experimental group. Box extends from the 25th to the 75th percentile with the median shown by the line in the middle. Whiskers are min to max with all data points shown.

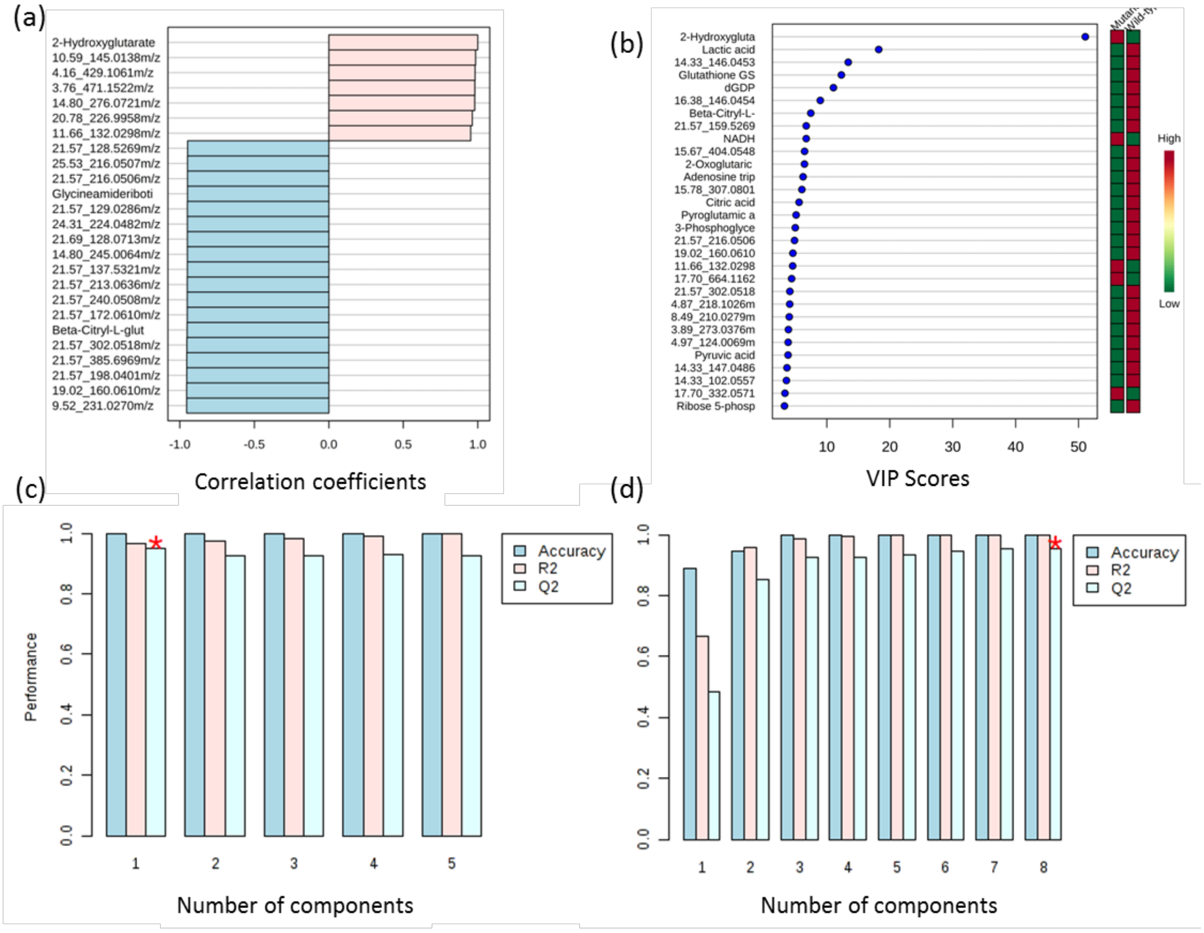

Supplementary Figure. 5 Univariate and multivariate statistical analysis of IDH mutant vs wild-type cells shows significant metabolic differences. (a) Top 25 compound features and annotated metabolites which correlat positively or negatively with the differences in 2-HG between mutant and wild-type cells. **(b)** Variable importance in the projection plot of top 30 annotated compound features and identified metabolites derived from partial least squares-discriminant analysis (PLS-DA) multivariate statistical analysis. Coloured boxes to right hand side of each metabolite qualitatively indicate degree and direction of change in abundance between the two experimental groups. **(c)** Performance of the PLS-DA model showed that a 1 component model (red star, using only 2-hydroxyglutarate) provided very high accuracy and validation (R^2, Q^2 and accuracy were predicted using ‘Leave one out’ cross validation (R^2=0.99; Q^2=0.92; Accuracy=100%). **(d)** Without 2-hydroxyglutarate an 8 component model differentiates IDH1 mutant from wild type cells (R^2=0.99, Q^2=0.96 and accuracy >0.99; predicted using ‘leave one out’ cross validation).

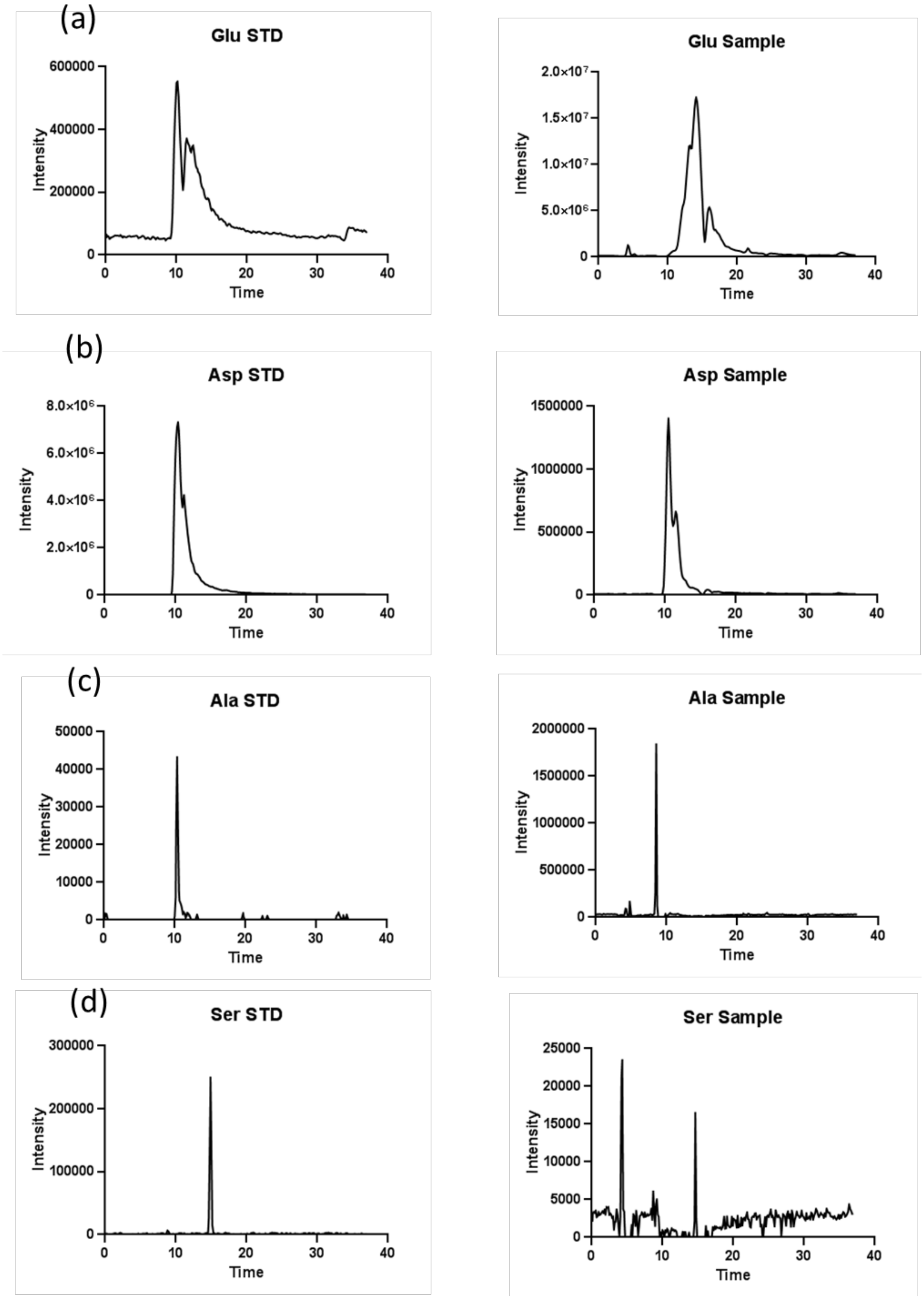

Supplementary Figure 6: EICs for four proteinogenic amino acids which showed a chromatographic peak from both authentic standards and cell extracts. (a) EIC from IC-MS analysis of glutamate from authentic standard and IDH mutant cell extract. **(b)** EIC from IC-MS analysis of aspartate from authentic standard and IDH mutant cell extract. **(c)** EIC from IC-MS analysis of alanine from authentic standard and IDH mutant cell extract. **(d)** EIC from IC-MS analysis of serine from authentic standard and IDH mutant cell extract. All extracted ion chromatograms used a <5ppm accurate mass cut-off.

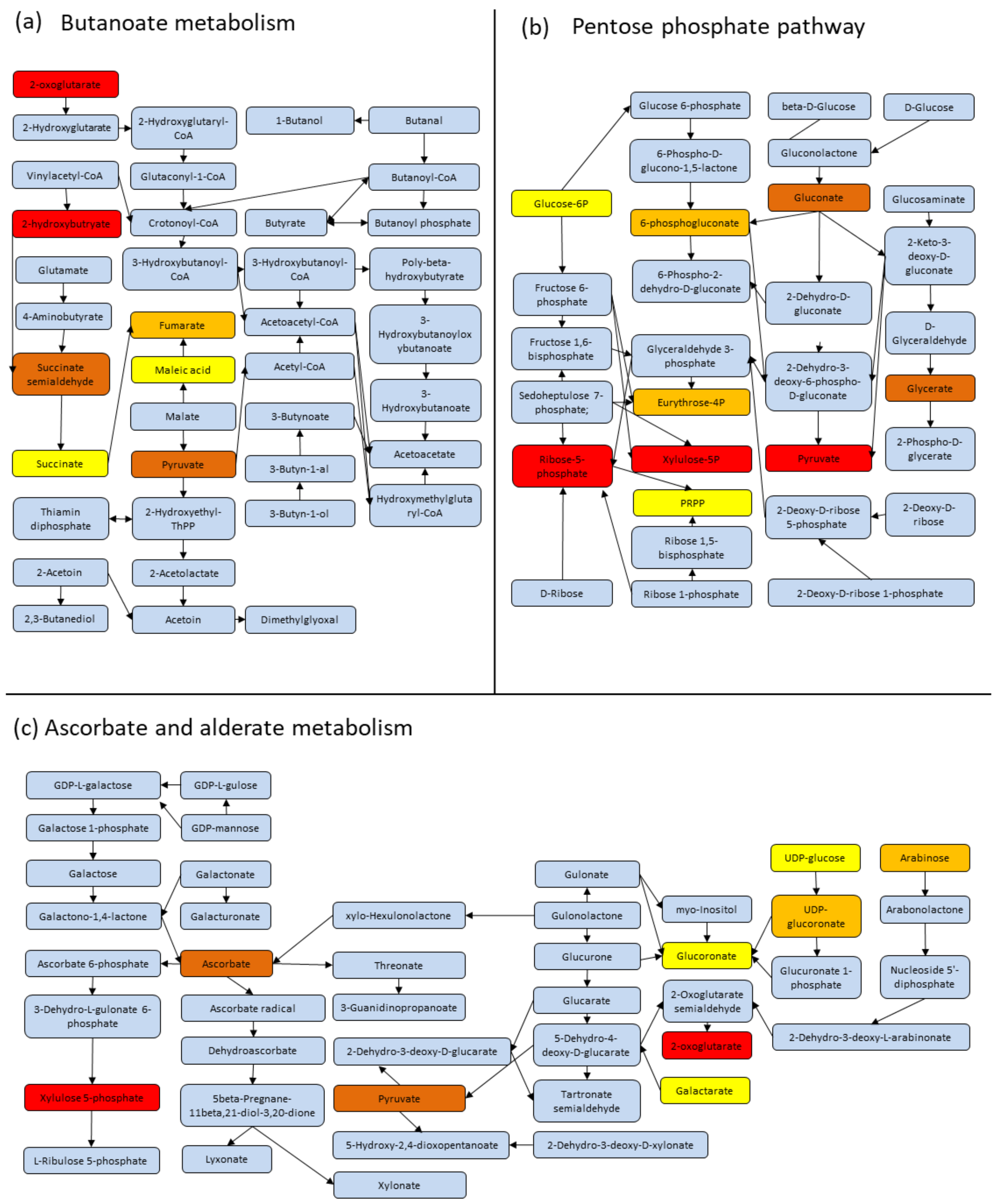

Supplementary Figure 7: Pathways analysis. **(a)** Butanoate metabolism (false discovery rate (FDR) adjusted p-value = 1.01E-08; impact = 0.2). **(b)** Pentose Phosphate Pathway (FDR adjusted p-value = 4.04E-06; impact = 0.49). **(c)** Ascorbate and alderate metabolism (FDR adjusted p-value = 1.01E-08; impact = 0.17). Pathways enrichment analysis using MetaboAnalyst used the Kyoto Encyclopaedia of Genes and Genomes (KEGG) pathway library for Homo sapiens reproduced here. Blue KEGG code = not identified in dataset but used for background to enrichment analysis; yellow-orange-red graded = identified in dataset with low-medium-high significance respectively.

(a) Glycoxylate metabolism

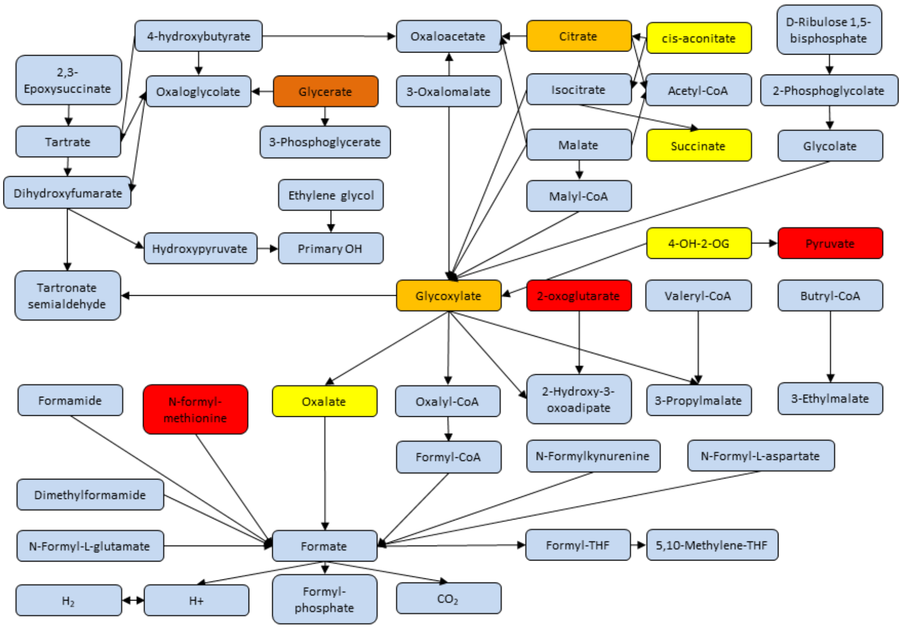

(b) TCA cycle

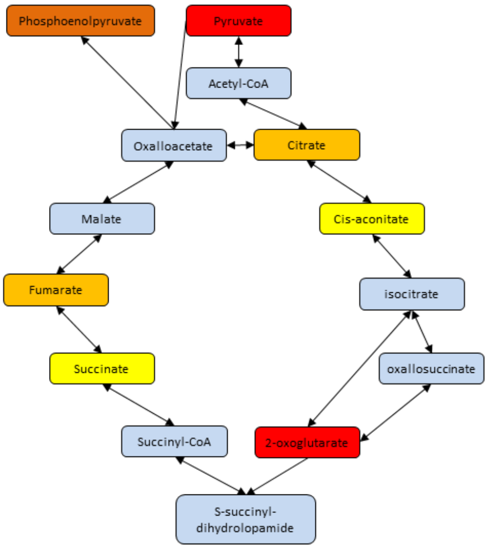

Supplementary Figure 8: Pathways analysis continued. **(a)** Glycoxylate metabolism (FDR adjusted p-value = 5.47E-06; impact = 0.4). **(b)** TCA cycle (FDR adjusted p-value = 5.47E-06; impact = 0.32). Although only two metabolites were identified in the tryptophan and lysine degradation pathways both pathways were predicted to be significantly altered; lysine (FDR adjusted p-value = 8.23E-10; impact = 0.01) and tryptophan (FDR adjusted p-value = 8.23E-10; impact = 0.02). These pathways are not shown here due to relatively small number of identified metabolites. Pathways enrichment analysis using MetaboAnalyst used the KEGG pathway library for Homo sapiens reproduced here. Blue KEGG code = not identified in dataset but used for background to enrichment analysis; yellow-orange-red graded = identified in dataset with low-medium-high significance respectively.
