# Supplementary material for: Anion-exchange chromatography mass spectrometry provides extensive coverage of primary metabolic pathways revealing altered metabolism in IDH1 mutant cells
Source: Commun Biol. 2020 May 20;3:247. doi: 10.1038/s42003-020-0957-6 (PMC7239943; doi:10.1038/s42003-020-0957-6)
Supplement: Supplementary file 3 — Reporting Summary [file 42003_2020_957_MOESM3_ESM.pdf]

natureresearch

Corresponding author(s): James McCullagh

Last updated by author(s): 2020/04/14

Reporting Summary

Nature Research wishes to improve the reproducibility of the work that we publish. This form provides structure for consistency and transparency in reporting. For further information on Nature Research policies, see Authors & Referees and the Editorial Policy Checklist.

Statistics

For all statistical analyses, confirm that the following items are present in the figure legend, table legend, main text, or Methods section.

n/a	Confirmed	
☐	☒	The exact sample size (*n*) for each experimental group/condition, given as a discrete number and unit of measurement
☐	☒	A statement on whether measurements were taken from distinct samples or whether the same sample was measured repeatedly
☐	☒	The statistical test(s) used AND whether they are one- or two-sided<br>*Only common tests should be described solely by name; describe more complex techniques in the Methods section.*
☐	☒	A description of all covariates tested
☐	☒	A description of any assumptions or corrections, such as tests of normality and adjustment for multiple comparisons
☐	☒	A full description of the statistical parameters including central tendency (e.g. means) or other basic estimates (e.g. regression coefficient) AND variation (e.g. standard deviation) or associated estimates of uncertainty (e.g. confidence intervals)
☐	☒	For null hypothesis testing, the test statistic (e.g. *F*, *t*, *r*) with confidence intervals, effect sizes, degrees of freedom and *P* value noted<br>*Give P values as exact values whenever suitable.*
☒	☐	For Bayesian analysis, information on the choice of priors and Markov chain Monte Carlo settings
☒	☐	For hierarchical and complex designs, identification of the appropriate level for tests and full reporting of outcomes
☐	☒	Estimates of effect sizes (e.g. Cohen's *d*, Pearson's *r*), indicating how they were calculated

Our web collection on statistics for biologists contains articles on many of the points above.

Software and code

Policy information about availability of computer code

Data collection	Xcaliber and Chromeleon (ThermoFisher Scientific)
Data analysis	Progenesis QI (Non-Linear Dynamics; MetaboAnalyst;

For manuscripts utilizing custom algorithms or software that are central to the research but not yet described in published literature, software must be made available to editors/reviewers. We strongly encourage code deposition in a community repository (e.g. GitHub). See the Nature Research guidelines for submitting code & software for further information.

Data

Policy information about availability of data

All manuscripts must include a data availability statement. This statement should provide the following information, where applicable:

- Accession codes, unique identifiers, or web links for publicly available datasets
- A list of figures that have associated raw data
- A description of any restrictions on data availability

The summary statistics and metadata generated from the untargeted mass spectrometry experiments reported in this study (referred to as Batch 1 and Batch 2) are available in Tables 1-5 and the Supplementary Figures and Tables. The raw mass spectrometry data files from which the results were generated have been deposited in Metabolites (Reference: MTBLS1654) a freely accessible public data archive.

Field-specific reporting

Please select the one below that is the best fit for your research. If you are not sure, read the appropriate sections before making your selection.

☒ Life sciences ☐ Behavioural & social sciences ☐ Ecological, evolutionary & environmental sciences

For a reference copy of the document with all sections, see nature.com/documents/nr-reporting-summary-flat.pdf

Life sciences study design

All studies must disclose on these points even when the disclosure is negative.

Sample size	The number of biological cell sample replicates in this study was determined by experience. Given the exploratory nature of untargeted metabolomics, where, by definition a large range of analytes are of interest across a wide concentration range that likely exceeds the dynamic range of the analytical system, it is not possible to calculate the number of replicates required. 6 and 9 tissue cultures replicates were used for each experimental group. This sample size was determined through trial an error.
Data exclusions	In general no data was excluded from the datasets discussed in this study unless explicitly described in the text.
Replication	As described in the manuscript the studies presented were replicated on a number of occasions over a 18 month period. The replicate data and its discussion is presented in the manuscript.
Randomization	All sample analyses were randomized
Blinding	Once data was generated by the mass spectrometer systems it was not blinded to the analyst. It was necessary for the data processing and statistical analysis to have knowledge of the experimental groups.

Reporting for specific materials, systems and methods

We require information from authors about some types of materials, experimental systems and methods used in many studies. Here, indicate whether each material, system or method listed is relevant to your study. If you are not sure if a list item applies to your research, read the appropriate section before selecting a response.

Materials & experimental systems

n/a	Involved in the study	
☒	☐	Antibodies
☐	☒	Eukaryotic cell lines
☒	☐	Palaeontology
☒	☐	Animals and other organisms
☒	☐	Human research participants
☒	☐	Clinical data

Methods

n/a	Involved in the study	
☒	☐	ChIP-seq
☒	☐	Flow cytometry
☒	☐	MRI-based neuroimaging

Eukaryotic cell lines

Policy information about cell lines

Cell line source(s)	Cell lines were purchased from ATCC
Authentication	Authentication from ATCC was relied upon, none of the cell lines were further authenticated
Mycoplasma contamination	Cell lines were regularly tested for mycoplasma contamination, all tests were negative.
Commonly misidentified lines (See ICLAC register)	none
