# Supplementary material for: Anion-exchange chromatography mass spectrometry provides extensive coverage of primary metabolic pathways revealing altered metabolism in IDH1 mutant cells
Source: Commun Biol. 2020 May 20;3:247. doi: 10.1038/s42003-020-0957-6 (PMC7239943; doi:10.1038/s42003-020-0957-6)
Supplement: Supplementary file 4 — Description of Additional Supplementary Files [file 42003_2020_957_MOESM4_ESM.pdf]

Description of Additional Supplementary Files

File Name: **Supplementary Data 1**
Description: Metabolite standards database including associated measurement parameters used for identification in unknown samples. Authentic standards were included in the list when >1000 ions associated with a discrete chromatographic retention time were obtained from a 5uL injection of a 1ug/mL standard in 80% MeOH [See separate Excel File]

File Name: **Supplementary Data 2**
Description: LN18 IDH mutant vs wild type cells: Identified compound features, measurement parameters and statistics (Batch 1). [See separate Excel File]

File Name: **Supplementary Data 3**
Description: Volcano plot data LN18 IDH mutant vs wild type [See separate Excel File]

File Name: **Supplementary Data 4**
Description: IDH mutant vs wild-type cell dataset (Batch 2) [See separate Excel File]

File Name: **Supplementary Data 5**
Description: Pathways Analysis statistics (Batch 2) [See separate Excel File]
